# Clinical Applications of Artificial Intelligence in Medical Imaging and Image Processing—A Review

**DOI:** 10.3390/cancers16101870

**Published:** 2024-05-14

**Authors:** Rafał Obuchowicz, Michał Strzelecki, Adam Piórkowski

**Affiliations:** 1 Department of Diagnostic Imaging, Jagiellonian University Medical College, 31-008 Krakow, Poland; rafalobuchowicz@su.krakow.pl; 2 Institute of Electronics, Lodz University of Technology, 93-590 Lodz, Poland; 3 Department of Biocybernetics and Biomedical Engineering, AGH University of Krakow, 30-059 Krakow, Poland

## 1. Introduction

Artificial intelligence (AI) is currently becoming a leading field in data processing. While the results it produces are spectacular when it comes to simple statistical relationships, in the case of medical imaging, the search for optimal methods is still ongoing. This collection of articles [1,2,3,4,5,6,7,8,9,10,11,12,13,14,15,16,17,18,19,20,21,22,23,24,25,26,27,28,29,30,31,32,33,34] focuses on the Topic “Artificial Intelligence in Medical Imaging and Image Processing” and discusses the latest achievements in this area.

Artificial intelligence refers to the simulation of human intelligence in machines that are programmed to think and learn like humans. It involves the development of algorithms and computer systems that can perform tasks which typically require human reasoning, adaptability, and simulation of cognitive processes. AI encompasses a broad range of techniques and technologies aimed at creating systems capable of reasoning, problem-solving, perception, and learning. AI has found various applications in medical imaging [35,36,37], revolutionizing the field by enhancing diagnostic accuracy, efficiency, and patient care. Some important applications of AI in medical imaging are as follows:

− Image segmentation—identification and delineation of specific structures or regions of interest within medical images;
− Disease detection and diagnosis—identification of abnormalities by highlighting potential areas of concern; early detection and diagnosis of various medical conditions by analyzing medical images regardless of imaging modality;
− Image preprocessing—enhancing the quality of medical images and reconstructing from incomplete or noisy data to improve their overall clarity and diagnostic value;
− Personalized treatment planning—tailoring treatment plans based on individual patient characteristics and response to therapy;
− Predictive analytics—analysis of medical imaging data along with other clinical information to predict disease progression, treatment response, and potential complications, enabling better-informed decision-making;
− Quality control—maintaining the quality of medical images by detecting artifacts, ensuring that images used for diagnosis are of the highest quality;
− Monitoring and follow-Up—assisting in the continuous monitoring of disease progression and treatment response over time, enabling timely adjustments to the treatment plan.

Currently, there are two main groups of AI-based approaches which are widely used in medical imaging. The first group encompasses machine learning (ML), i.e., algorithms that enable a system to learn independently based on data. The other approach, which in fact is a subset of ML, involves using complex artificial neural networks to model and solve problems. The main difference between these groups lies in the fact that ML models require the definition of a set of features that characterize the objects under analysis. In the case of analyzing medical images, radiomics is a significant source of such features. Radiomics is a field of study within medical imaging that involves the extraction and analysis of quantitative features from medical images. These features are derived from the analysis of intensity, shape, texture, and other characteristics present in medical data [38]. In particular, the analysis of image texture provides a wealth of information about the morphology and physiology of organs and tissues. Texture analysis is an effective tool that provides machine learning models with diagnostically relevant parameters obtaied from images of various modalities [39]. In contrast to classical machine learning models, deep networks are capable of determining significant parameters of the examined organs on their own within their internal convolutional layers. The input for such algorithms are images, and the output is the response to a given research problem (e.g., a segmented image or the classification of a pathological change). Based on an analysis of the literature, it can be stated that, currently, deep learning algorithms are more prevalent than classical ML methods. Usually, deep learning provides more accurate and reliable outcomes. There are, however, exceptions. For example, in [40], it was demonstrated that the texture analysis of selected vertebrae in CT images provided a more accurate prediction of a patient’s age than deep learning methods.

This collection includes eight articles on detection, five on classification, four on segmentation, seven on prediction, five on quality improvement, and three on simulation. The following organs were of interest: the lungs (4), breasts (4), liver (3), brain (9), prostate (3), and others (8). Two studies were conducted on phantoms. The most popular imaging modalities are MRI (11) and CT (8), but there are also works on radiography, ultrasound, and microscopic images. The achieved efficiencies cannot be clearly compared due to different research methodologies, but, as an example, in **detection tasks, efficiency ranged from approx. 65% to even 100%**. The exact results can be found in the relevant table (Appendix A).

## 2. Review of AI Applications in Medical Imaging

### 2.1. Deep Learning for Detecting Brain Metastases on MRI: A Systematic Review and Meta-Analysis

In this investigation, a systematic review and meta-analysis were undertaken to evaluate the diagnostic efficacy of deep learning algorithms utilizing magnetic resonance imaging (MRI) for the identification of cerebral metastases in oncological patients. The methodology encompassed a comprehensive literature search across MEDLINE, EMBASE, and Web of Science, culminating on 30 September 2022 and adhering to pre-specified inclusion and exclusion criteria to ensure the selection of relevant original research articles. The inclusion criteria mandated the investigation of patients with brain metastases using deep learning models applied to MRI data, with a clear articulation of diagnostic performance metrics. Conversely, the exclusion criteria filtered out non-original articles, small case series, studies with overlapping patient cohorts, and papers with insufficient details on diagnostic performance.

This qualitative synthesis of 24 studies, following rigorous quality assessment via the Quality Assessment of Diagnostic Accuracy Studies-2 (QUADAS-2) and the Checklist for Artificial Intelligence in Medical Imaging, yielded a pooled detectability rate of 89% at both the patient and lesion levels. This synthesis underscores the potent utility of deep learning paradigms in enhancing the precision of cerebral metastases detection, thereby facilitating timely and informed clinical decision-making.

The findings advocate for stringent adherence to established reporting standards within the domain of artificial intelligence in medical imaging in order to augment the reproducibility and interpretability of diagnostic accuracy studies. The evident heterogeneity in reported false-positive rates precluded a unified analysis, highlighting an area for future standardization. Collectively, this study elucidates the significant potential of deep learning models in advancing our diagnostic capabilities in the field of brain metastases, thereby potentially ameliorating patient management and prognostic outcomes in the oncological setting.

### 2.2. Extended Reality in Diagnostic Imaging—A Literature Review

The past decade has witnessed an unprecedented surge in the adoption of Extended Reality (ER) in healthcare, with a particular focus on its applications in diagnostic imaging, patient positioning, and medical education. A comprehensive analysis of scientific publications was conducted to explore ER’s potential benefits and utilization in various medical contexts, including ultrasound, interventional radiology, and computed tomography. Additionally, this study investigated ER’s role as a potential alternative to traditional anesthesia and sedation during medical examinations.

The integration of ER into medical education has garnered significant attention due to its ability to create immersive learning environments, particularly in anatomy and patient positioning. However, questions have arisen regarding the economic feasibility of investing in ER technology and its ongoing maintenance costs.

Overall, the analysis suggests that implementing ER in clinical practice holds promise for expanding diagnostic capabilities, improving medical education outcomes, and enhancing patient experiences through increased visualization and comprehension of medical conditions. While further research is needed to fully realize ER’s potential in healthcare and address associated challenges, its adoption represents an innovative approach to advancing diagnostic imaging, medical education, and patient care.

### 2.3. Redefining Radiology: A Review of Artificial Intelligence Integration in Medical Imaging

This review delves into the integration of artificial intelligence (AI) into radiology, highlighting its transformative impact on healthcare. It covers the evolution from traditional X-ray discovery to the adoption of machine learning and deep learning for medical image analysis. The key AI applications it discusses include image segmentation, computer-aided diagnosis, predictive analytics, and workflow optimization, all contributing to improved diagnostic accuracy, personalized treatment, and increased clinical efficiency. Despite these benefits, challenges such as data integrity, algorithm transparency, and ethical concerns are acknowledged. The review concludes optimistically, advocating for ongoing research, technological advancement, and collaboration between radiologists and AI developers to harness AI’s full potential in radiology, underscoring the importance of innovation and ethical responsibility.

## 3. Modality: X-ray

### 3.1. Key-Point Detection Algorithm of Deep Learning Can Predict Lower Limb Alignment with Simple Knee Radiographs

This study leveraged a convolutional neural network (CNN) to predict the weight-bearing line (WBL) ratio from knee anteroposterior (AP) radiographs, enhancing the diagnosis of conditions like osteoarthritis. Employing stratified random sampling, this study analyzed 4790 knee AP radiographs from 2410 patients, using annotated and cropped images to focus the CNN’s analysis. The CNN model’s key-point detection capability to identify crucial tibial plateau points facilitated accurate WBL ratio calculations. This method demonstrated an accuracy comparable to that of direct measurements from full-leg radiographs and promises significant benefits for the medical community and patients by offering a more precise, efficient, and accessible tool for assessing knee alignment, potentially improving the early detection and treatment of related conditions.

### 3.2. Dynamic Chest Radiograph Simulation Technique with Deep Convolutional Neural Networks: A Proof-of-Concept Study

This research study introduces an innovative Radiograph Motion Simulation (RMS) network which integrates U-Net with LSTM networks to simulate and predict respiratory lung motion from single-phase chest X-rays. A Spatial Transformer Network is also applied for precise image deformation, reflecting true respiratory motion. The network’s performance is evaluated through qualitative and quantitative methods, including Dice score calculations for motion prediction accuracy. This approach enhances diagnostic capabilities by providing insights into lung dynamics from static X-rays, offers a non-invasive alternative for lung function assessment, and increases diagnostic efficiency by extracting detailed information from routine chest X-rays. Such advancements hold significant potential for improving pulmonary diagnostics, patient comfort, and healthcare efficiency.

### 3.3. Chest X-ray Foreign Objects Detection Using Artificial Intelligence

This study leveraged a deep convolutional neural network to identify objects in chest X-ray images using data from the NIH Chest X-ray Dataset. The employed techniques included manual image review for object identification, computer-assisted annotations, and preprocessing steps like resizing and normalization. The object detection model, based on the YOLOv8 architecture and Ultralytics framework, demonstrated an average precision of 0.815 in detecting foreign objects. This AI model presents several benefits for patients, including improved diagnostic accuracy, increased efficiency in processing X-ray images, support for radiologists by automating routine detections, and the potential for earlier disease detection, thereby enhancing patient care and treatment outcomes.

## 4. Modality: MRI

### 4.1. A Comparative Study of Automated Deep Learning Segmentation Models for Prostate MRI

This study focuses on enhancing prostate cancer diagnoses through advanced imaging techniques. It involves a comparative analysis of various segmentation models for the accurate delineation of the prostate gland and its zones, coupled with an innovative approach incorporating an object detection algorithm to aid the segmentation process. Conducted on two public datasets, this study finds that while most models yield similar results, the nnU-Net framework stands out for its superior performance. Additionally, models augmented with object detection preprocessing show enhanced generalizability. This research study offers significant benefits for medical practitioners, including improved diagnostic accuracy, better treatment planning, early detection, and increased efficiency, ultimately leading to better patient outcomes and quality of life.

### 4.2. A Deep Learning Radiomics Nomogram to Predict Response to Neoadjuvant Chemotherapy for Locally Advanced Cervical Cancer: A Two-Center Study

This study introduced a deep learning radiomics nomogram (DLRN) leveraging multiparametric MR imaging to predict responses to neoadjuvant chemotherapy (NACT) in locally advanced cervical cancer (LACC) patients. By extracting and analyzing both hand-selected and deep learning-based radiomics features from MR sequences, this study developed DLRN-integrated predictive radiomics signatures along with clinical features. Validated through AUC, calibration curves, and Decision Curve Analysis, the DLRN demonstrated superior predictive accuracy over traditional clinical models and radiomics signatures, highlighting its potential for personalizing treatment plans and improving prognostication. This approach supports doctors by enabling more informed treatment decisions and optimizing resource use, while patients benefit from tailored therapies potentially leading to better outcomes and reduced unnecessary treatments.

### 4.3. Radiologic vs. Segmentation Measurements to Quantify Wilms Tumor Volume on MRI in Pediatric Patients

This study explored the accuracy of measuring tumor volume in Wilms tumor patients using manual segmentation and traditional radiological methods and investigated the potential of automating the segmentation process with deep learning (nnU-Net). Manual segmentation typically resulted in larger tumor volume estimates compared to radiological measurements, highlighting a potential underestimation of tumor size when using traditional methods. nnU-Net-based automated segmentation achieved high accuracy, with a median Dice coefficient of 0.90 and a median 95th percentile Hausdorff distance of 7.2 mm. This suggests that deep learning can offer precise volume measurements efficiently while avoiding the time and observer variabilities associated with manual methods. Such advancements could lead to more accurate and personalized treatment planning, benefiting both the medical community and patients by enabling better treatment outcomes and potentially less invasive treatment options.

### 4.4. Textural Features of MR Images Correlate with an Increased Risk of Clinically Significant Cancer in Patients with High PSA Levels

This study employs a comprehensive approach to improve prostate cancer detection by combining multiparametric MRI guided by the PIRADS protocol with advanced image analysis techniques. Key methods include the manual delineation of prostate zones and tumor foci, extraction of over 7000 textural features using MaZda software (http://www.eletel.p.lodz.pl/programy/mazda/, accessed on 18 March 2024), and statistical analysis to correlate these features with PSA levels. The use of MIL-SVM machine learning further enhances diagnostic accuracy. This integrative approach yields a 92% accuracy rate in identifying prostate cancer, offering significant benefits such as improved diagnostic precision, early detection capabilities, personalized treatment options, a reduced need for invasive procedures, and optimized healthcare resources. This advancement in diagnostic technology holds promise for both the medical community and patients, potentially transforming prostate cancer diagnosis and treatment.

### 4.5. ‘Earlier than Early’ Detection of Breast Cancer in Israeli BRCA Mutation Carriers Applying AI-Based Analysis to Consecutive MRI Scans

This study developed an artificial intelligence (AI)-based approach, specifically a convolutional neural network (CNN), to improve the classification of enhancing foci in MRI scans of BRCA pathogenic variant carriers, aiming to reduce false-negative interpretations in breast cancer (BC) detection. The method involved manually segmenting retrospectively identified enhancing foci from previous MRIs, which were then used to train the CNN for accurate differentiation between malignant and benign/normal findings. This AI model successfully identified 65% of cancerous foci, particularly excelling in the detection of triple-negative BC. The application of this technology promises to enhance early BC detection in high-risk individuals, reduce diagnostic errors, and pave the way for more personalized and effective surveillance strategies, thereby offering significant benefits to the medical community in terms of improved patient care and outcomes.

### 4.6. Deep Learning Algorithm for Differentiating Patients with a Healthy Liver from Patients with Liver Lesions Based on MR Images

In this study, advanced diagnostic methodologies were employed to address the complexities of liver disease diagnosis using multiparametric magnetic resonance (MR) imaging. The core of this approach involved a sophisticated multiclass segmentation algorithm designed to differentiate between various types of liver lesions and healthy tissue. This comprehensive algorithmic pipeline, from data preprocessing to final classification, was rigorously evaluated using statistical metrics, notably the area under the receiver operating characteristic (AUC ROC) curve, demonstrating high accuracy in distinguishing between healthy and diseased liver states. This study’s findings offer significant benefits to patients, including the potential for early disease detection, improved diagnostic precision, and the standardization of liver disease assessments. These advancements promise to enhance patient outcomes through more timely and accurate treatment interventions in hepatology.

### 4.7. Performance of Fully Automated Algorithm Detecting Bone Marrow Edema in Sacroiliac Joints

This study utilized advanced MRI techniques and an automated algorithm to detect bone marrow edema (BME) in axial spondyloarthritis (axSpA) patients, focusing on the sacral and iliac bones. Key methods included MRI examination, deviation angle measurement for image accuracy, manual and automated bone segmentations, and BME lesion assessment using the SPARCC scale. The algorithm’s performance was evaluated through sensitivity and specificity analysis, showing high accuracy regardless of image acquisition angles.

The benefits of the methodology developed in this study are significant from a patient care perspective. Such methodology enhances diagnostic accuracy and efficiency, provides consistent results, enables early detection of axSpA, supports large-scale patient screening, and serves as an educational tool for clinicians. This approach promises to streamline the diagnostic process, improve patient outcomes, and foster a more efficient workflow in clinical settings.

### 4.8. Effects of Path-Finding Algorithms on the Labeling of the Centerlines of Circle of Willis Arteries

This study focused on the automated identification of centerlines in intracranial vessel segments using path-finding algorithms applied to 3D time-of-flight MRA images. Three algorithms were compared: depth-first search, Dijkstra’s, and A*. Among 840 vessel segments, Dijkstra’s algorithm exhibited the highest accuracy (97.1%), closely followed by the A* algorithm (96.1%), with the depth-first search algorithm showing lower accuracy (83.5%). This study highlighted Dijkstra’s and A* algorithms as effective and comparable in both accuracy and speed for delineating pathways in the circle of Willis arteries, offering significant benefits for medical imaging analysis in terms of diagnostic accuracy, treatment planning efficiency, and standardization of vascular imaging assessments.

### 4.9. Retrospective Motion Artifact Reduction by Spatial Scaling of Liver Diffusion-Weighted Images

This study introduced a data-driven algorithm to counteract motion-induced signal loss in diffusion-weighted MRI (DWI) of the liver. This algorithm enhances DWI by excluding heavily distorted images and applying spatially variable image scaling, based on a signal-loss model, to improve signal uniformity and accuracy, especially in the left lobe, which is most commonly affected by this phenomenon. Advantages for practitioners include improved diagnostic accuracy of liver DWI, more homogeneous liver imaging, accurate apparent diffusion coefficient (ADC) measurements, and expanded clinical utility of DWI for liver disease assessment.

### 4.10. Generating Synthetic Radiological Images with PySynthMRI: An Open-Source Cross-Platform Tool

PySynthMRI is an open-source, user-friendly software designed to create synthetic MR images with variable radiological contrasts by adjusting parameters like echo time, repetition time, and inversion time(s). It calculates pixelwise signal intensity from input images such as T1 and T2 maps and is compatible with Linux, Windows, and MacOS. The tool supports customization, allowing users to add new features and contrasts, and can export images in DICOM or NiFTI formats. In terms of its benefits for the medical community and for patients, PySynthMRI streamlines imaging by reducing scan times, enhances flexibility for both research and clinical applications, and creates the potential for enhanced diagnostic accuracy, making it a valuable resource for improving patient care and advancing medical research.

## 5. Modality: SPECT

### Convolutional Neural Networks to Classify Alzheimer’s Disease Severity Based on SPECT Images: A Comparative Study

This study employed convolutional neural networks (CNNs) and single-photon emission computed tomography (SPECT) imaging to analyze the progression of Alzheimer’s disease (AD). Utilizing a range of CNN models, from lightweight (MobileNet V2, NASNetMobile) to heavier models (VGG16, Inception V3, ResNet), this research study aimed to enhance the accuracy of AD diagnosis and early detection by analyzing complex patterns in neuroimaging data. The effectiveness of transfer learning was also demonstrated in this context, highlighting its potential to efficiently leverage limited datasets in medical imaging. This methodology offers a number of benefits for medical practitioners and for patients, including improved diagnostic accuracy, early detection, personalized treatment plans, optimized resources, and reduced cognitive assessment burden. This approach is a promising advancement in neurodegenerative disease diagnostics and patient care.

## 6. Modality: CT

### 6.1. COVID-19 and Cancer: A Complete 3D Advanced Radiological CT-Based Analysis to Predict the Outcome

Utilizing a retrospective cohort design, this study scrutinized computed tomography (CT)-derived pulmonary morphological alterations in 22 oncological subjects post SARS-CoV-2 infection. Employing quantitative metrics, such as Hounsfield Units for tissue density and volumetric analyses for pulmonary capacity, alongside three-dimensional radiometric evaluations, this study delineated significant post-infectious pulmonary sequelae characterized by fibrotic densification and volumetric reduction. These findings elucidate the exacerbation of pulmonary vulnerability in the oncological demographic when confronted with SARS-CoV-2, thereby underscoring the imperative for bespoke therapeutic stratagems. This research study contributes to the corpus of evidence-based medicine, facilitating optimized clinical decision-making and prognostication for oncological patients amidst the COVID-19 pandemic.

### 6.2. Using Deep-Learning-Based Artificial Intelligence Technique to Automatically Evaluate the Collateral Status of Multiphase CTA in Acute Ischemic Stroke

This study utilized multiphase computed tomography angiography (mCTA) and convolutional neural network (CNN) techniques to develop an AI model for the automatic prediction of collateral status in acute ischemic stroke patients. The AI approach aimed to enhance the efficiency and accuracy of collateral evaluation, traditionally performed through time-consuming manual assessments. This study demonstrated that CNN-based AI models could provide rapid and consistent evaluations of mCTA images, offering significant benefits for both clinicians and patients. For doctors, this method reduces the workload and enables quicker decision-making, which is crucial in stroke management. Patients stand to benefit from timely and personalized treatment interventions, potentially improving recovery outcomes and reducing the risk of long-term disability.

### 6.3. Sinogram Inpainting with Generative Adversarial Networks and Shape Priors

This study introduces a novel method utilizing generative adversarial networks (GANs) to enhance X-ray computed tomography (CT) image reconstruction, particularly in cases with limited tomographic measurements due to incomplete scanning coverage. This method integrates shape priors to accurately infer missing data, thereby reducing artifacts and improving image quality. Unlike traditional approaches that address the interpolation of evenly spaced missing angles, this method is adept at reconstructing images with substantial gaps in consecutive scanning angles. The application of this technique has demonstrated significant improvements in image fidelity, evidenced by a 7 dB increase in Peak Signal-to-Noise Ratio (PSNR) compared to existing sinogram-inpainting methods. For medical professionals and patients, this advancement offers multiple benefits, including higher-quality images for more reliable diagnoses, increased efficiency of imaging procedures, and reduced patient exposure to ionizing radiation. This approach is a significant step forward in medical imaging, potentially transforming CT imaging practices by optimizing the use of limited or suboptimal datasets.

### 6.4. A Deep Learning Approach for Rapid and Generalizable Denoising of Photon-Counting Micro-CT Images

This study introduces UnetU, a deep learning model based on a 2D U-net convolutional neural network designed to denoise photon-counting CT (PCCT) images by approximating iterative reconstruction from weighted filtered backprojection (wFBP) images. UnetU employs a custom loss function and a specific transformation of wFBP to improve material decomposition accuracy across various energy thresholds. This method offers significant benefits to the medical community, including faster reconstruction times, improved image quality, enhanced material decomposition, broad applicability, and increased clinical workflow efficiency. UnetU’s potential to accelerate and refine PCCT imaging promises substantial advancements in both clinical diagnostics and preclinical research.

### 6.5. Segmentation of Portal Vein in Multiphase CTA Image Based on Unsupervised Domain Transfer and Pseudo Label

This study introduces an innovative approach for segmenting the portal vein in multiphase CT angiography (CTA) images, leveraging unsupervised domain transfer and pseudo labeling techniques. The process begins by stylistically aligning hepatic arterial phase (H-phase) and equilibrium phase (E-phase) images with portal vein phase (P-phase) images to mitigate contrast media discrepancies. Pseudo labels generated from P-phase annotations guide the segmentation of H-phase and E-phase images, resulting in accurate portal vein delineation across all phases.

This methodology presents several benefits for patient care: it provides a comprehensive diagnostic view by enabling portal vein analysis across multiple imaging phases, enhances efficiency by reducing the need for manual annotation, and improves segmentation accuracy through style normalization. Furthermore, it optimizes resource use by extending the utility of existing annotations and ultimately supports improved patient care through more precise diagnostic insights.

### 6.6. Deep Learning-Based vs. Iterative Image Reconstruction for Unenhanced Brain CT: A Quantitative Comparison of Image Quality

This study investigated image quality in brain CT scans using two reconstruction algorithms: the iterative AIDR-3D and the deep learning-based AiCE. Through a preliminary phantom study and a retrospective analysis of 100 emergency brain CTs, it assessed image noise, artifact presence, and contrast-to-noise ratios. The findings revealed AiCE’s superiority in reducing image noise and enhancing contrast-to-noise ratios, while AIDR-3D showed lower artifact indices. These insights into the differential benefits of each algorithm can aid patient care, optimizing CT imaging for more accurate diagnoses by offering enhanced image quality, better visualization of brain structures, and informed choices in algorithm selection for clinical practice.

### 6.7. Image Quality Improvement in Deep Learning Image Reconstruction of Head Computed Tomography Examination

This study compared the image quality of cranial CT scans reconstructed using deep learning image reconstruction (DLIR) with those using adaptive statistical iterative reconstruction (ASIR-V). Through both objective measurements (SNR and CNR in the brain’s grey and white matter) and subjective evaluations by experienced radiologists, DLIR demonstrated superior image quality, showing significant improvements in SNR (up to 54% for grey matter and 60% for white matter) and CNR (58% in BGA and 50% in PCF) compared to ASIR-V. Subjectively, DLIR also received higher rating scores from radiologists. This suggests that DLIR offers notable advantages in enhancing cranial CT scan quality, potentially improving diagnostic accuracy and patient care in medical imaging.

## 7. Modality: Ultrasonography

### Use of Automated Machine Learning for Classifying Hemoperitoneum on Ultrasonographic Images of Morrison’s Pouch: A Multicenter Retrospective Study

This study explored the use of automated machine learning (AutoML) for identifying the presence of hemoperitoneum in ultrasonography (USG) images of Morrison’s pouch in trauma patients. Utilizing a dataset of 2200 USG images from 864 patients across multiple South Korean trauma centers, this research study employed Google’s open-source AutoML for model training and validation. The process involved training the model with 1800 images, internal validation with 200 images, and external validation with an additional 200 images from an independent center. The AutoML model demonstrated high accuracy, with sensitivity and specificity rates exceeding 94% in both internal and external validations and an area under the receiver operating characteristic (AUROC) curve of 0.97. These results highlight AutoML’s potential to efficiently and accurately classify medical images, offering significant benefits for healthcare professionals, such as improved diagnostic reliability, efficiency in emergency care, scalability, and accessibility, thereby promising to enhance patient care in trauma and emergency settings.

## 8. Modality: Mammography

### 8.1. Avoiding Tissue Overlap in 2D Images: Single-Slice DBT Classification Using Convolutional Neural Networks

This study leverages digital breast tomosynthesis (DBT) and deep learning to enhance breast cancer screening, addressing the limitations of mammography, like tissue overlap. By modifying a deep learning framework with adjustments to the fully connected layers and regularization, as well as by employing data augmentation techniques to increase dataset variability, the model effectively classifies DBT slices as benign or malignant. Utilizing 2772 augmented images for training, the model achieved a 93.2% accuracy rate on the test set, with high sensitivity, specificity, precision, F1-score, and Cohen’s kappa values. These results underscore the potential of DBT coupled with deep learning to improve diagnostic accuracy, reduce false positives/negatives, and potentially surpass traditional mammography in terms of screening efficacy, offering substantial benefits to the medical community and patients.

### 8.2. Automated Computer-Assisted Medical Decision-Making System Based on Morphological Shape and Skin Thickness Analysis for Asymmetry Detection in Mammographic Images

This study employed Dynamic Time Warping (DTW) for morphological analysis and the Growing Seed Region (GSR) method for skin segmentation in mammographic images to detect asymmetries, which are indicative of potential breast cancer. DTW assesses the similarity between a patient’s two breasts by analyzing the distances from the breast image centroid to its perimeter, taking into account possible geometric distortions. GSR identifies skin-related asymmetries by expanding a seed set on skin pixels based on intensity and depth similarity. These methods enhance early cancer detection, increase diagnostic efficiency by speeding up the analysis process, improve accuracy in identifying asymmetries, and offer patient-specific insights, thereby supporting more personalized diagnostic and treatment approaches. Overall, the DTW and GSR methods significantly contribute to the early detection and diagnosis of breast cancer, offering substantial benefits to healthcare professionals in delivering effective patient care.

## 9. Other Imaging Techniques (Histological Analyses, Comparative Studies)

### 9.1. DBE-Net: Dual Boundary-Guided Attention Exploration Network for Polyp Segmentation

The Dual Boundary-guided Attention Exploration Network (DBE-Net) introduces innovative methods for polyp segmentation in colonoscopy, addressing key challenges such as indistinct boundaries, size variability, and resemblance to adjacent tissues. Incorporating a dual boundary-guided module, multi-scale enhancement, and low-level detail enhancement, DBE-Net significantly improves the precision of polyp detection. This advancement offers substantial benefits, including enhanced diagnostic accuracy, improved treatment outcomes, increased procedural efficiency, and reduced patient burden. Demonstrating superior performance on benchmark datasets, DBE-Net holds promise for advancing colorectal care by aiding in early and accurate polyp identification, contributing positively to both medical practice and the patient experience.

### 9.2. Application of Deep Learning Methods in a Moroccan Ophthalmic Center: Analysis and Discussion

This study introduces the application of two artificial intelligence (AI) methodologies—the U-Net algorithm for segmentation and the You Look Only Once Version 5 (YOLOv5) for detection—to diagnose diabetic retinopathy (DR) in color fundus images. U-Net is utilized to distinguish and color-code hemorrhages and exudates, enhancing visual assessment, while YOLOv5 detects these DR indicators, assigning a confidence score to each finding. These AI-driven approaches significantly benefit clinicians by improving diagnostic accuracy, increasing efficiency, enabling early detection of DR, reducing workload, and facilitating greater access to quality eye care. This study demonstrates that these AI tools can outperform traditional diagnostic methods, with the detection algorithm successfully identifying 100% of DR signs compared to lower detection rates by expert and resident doctors.

### 9.3. Deep Learning- and Expert Knowledge-Based Feature Extraction and Performance Evaluation in Breast Histopathology Images

This study explored three feature extraction methods for breast cancer diagnosis using histopathology images: a convolutional neural network (CNN), transfer learning with the VGG16 architecture, and a knowledge-based system. These methods were evaluated using seven classifiers on the BreakHis 400× image dataset. The CNN and VGG16 approaches showed promising results, with accuracies up to 85% and 86%, respectively, while the knowledge-based system outperformed them both, reaching an accuracy of up to 98%. These advancements have a significant impact on patient care, including improved diagnostic accuracy, increased efficiency in processing histopathology images, and the potential for standardizing diagnostic criteria. Such innovations hold the promise of enhancing breast cancer detection and treatment, thereby improving patient outcomes.

### 9.4. Deep-Learning-Based Dose Predictor for Glioblastoma–Assessing the Sensitivity and Robustness for Dose Awareness in Contouring

This study focused on developing a 3D VMAT dose prediction model for glioblastoma treatment using deep learning techniques, specifically cascaded 3D U-Nets. This model was trained and tested on a dataset of 125 glioblastoma patients, with the aim of improving the efficiency and quality of radiation therapy planning. The model demonstrated good sensitivity to realistic contour variations and was further refined for robustness against out-of-distribution cases. The successful implementation of this deep learning model in radiation therapy planning presents significant benefits for practitioners and for patients, including enhanced treatment planning efficiency, increased accuracy in dose predictions, and improved quality assurance in automated contouring processes, leading to more personalized and effective treatment strategies for patients.

### 9.5. Exploration and Enhancement of Classifiers in the Detection of Lung Cancer from Histopathological Images

This study utilized advanced computational methods to enhance lung cancer detection from histopathological images, employing techniques like enhanced Kernel Fuzzy C-Means segmentation, Particle Swarm, and Grey Wolf Optimization for dimensional reduction, and feature selection algorithms such as Kullback–Leibler Divergence and Invasive Weed Optimization. Seven classifiers, including SVM, KNN, and decision trees, were used for categorizing images, with hyperparameter tuning to improve accuracy. This approach offers significant benefits for medical professionals, including early cancer detection, higher diagnostic accuracy, efficiency in handling large image sets, objective assessments, personalized treatment planning, and educational opportunities in digital pathology.

### 9.6. Towards Realistic 3D Models of Tumor Vascular Networks

This study employed sophisticated computational techniques for the reconstruction and examination of neoplastic vascular architectures derived from histological sections. The methodological framework encompassed image registration algorithms, which were instrumental in rectifying spatial discrepancies across sequential histological slices, ensuring fidelity in the reconstitution of the three-dimensional vascular continuum. The procedure entailed a preliminary feature- and area-based alignment, succeeded by an exhaustive parallel registration for holistic dataset alignment. This study harnessed both intensity- and color-thresholding methods, supplemented by heuristic analyses, to precisely demarcate vascular entities within histological specimens. The capability to generate intricate three-dimensional models of tumor vasculature augments our comprehension of tumor biology, particularly of the spatial and morphological characteristics of vascular networks, which are pivotal in understanding tumor growth, metastasis, and angiogenesis. The detailed visualization of the vascular architecture within tumors aids in the strategic planning of diagnostic and therapeutic interventions, enabling clinicians to identify viable routes for targeted therapies or surgical excisions, thereby optimizing patient-specific treatment protocols.

### 9.7. Combining State-of-the-Art Pre-Trained Deep Learning Models: A Noble Approach for Skin Cancer Detection Using Max Voting Ensemble

This study implements a max voting ensemble technique, combining predictions from various pre-trained deep learning models, such as MobileNetV2, AlexNet, VGG16, ResNet50, DenseNet201, DenseNet121, InceptionV3, ResNet50V2, InceptionResNetV2, and Xception, for skin cancer classification. These models, pre-trained on skin cancer datasets, contribute individual predictions that are aggregated through max voting to achieve a final classification. This ensemble approach enhances diagnostic accuracy by leveraging the diverse strengths of each model, offering significant benefits to healthcare professionals. It ensures more reliable skin cancer classification, boosts confidence in diagnostic decisions, saves time in diagnosis, and provides robust support for complex cases, thereby improving patient care and treatment efficacy.

## Appendix A

**Table A1:** Summary of research objectives, analysed organs (locations), imaging modalities, methods, and results described in papers published in this collection.

No.	Objective	Localization	Imaging	Methods	Results
1	A systematic review of the performance of DL models that use MRI to detect BMs in cancer patients	Brain	MRI	25 DL algorithms	Deep learning algorithms effectively detect BMs with a pooled sensitivity of 89%
2	Outcome prediction for cancer patients infected with COVID-19	Lung	CT	3D radiometric analysis	COVID-19 infection may have further detrimental effects on the lungs of cancer patients
3	Prediction of the weight-bearing line (WBL) ratio using simple knee radiographs	Knee	X-ray	CNN	Proposed method for predicting lower limb alignment demonstrated comparable accuracy to that of direct measurement using whole-leg radiographs
4	Breast cancer detection using digital breast tomosynthesis	Breast	Digital breast tomosynthesis	CNN	Accuracy of 93.2%; sensitivity, specificity, precision, and F1-score of 92%, 94%, 94%, and 94%, respectively
5	Comparative study of different models for prostate segmentation	Prostate	MRI	Various segmentation deep networks	nnUNet gives most accurate segmentation results
6	New approach for polyp segmentation	Polyp	Endoscopic images	Dual boundary-guided attention exploration network (DBE-Net) for polyp segmentation	mDice of the proposed model reaches 82.4% and 80.6% for 2 datasets
7	Construction of a nomogram based on multiparametric MR images for predicting the response to neoadjuvant chemotherapy in patients with locally advanced cervical cancer	Cervix	MRI	Extraction of hand-selected (including texture) and DL-based radiomics features.	DL features slightly overlap with hand-selected ones in response prediction
8	Testing various CNN architectures to assess Alzheimer’s disease severity	Brain	SPECT	MobileNet V2, NASNetMobile, VGG16, Inception V3, and ResNet	Good results for all networks; the best was Resnet, with an average accuracy 65%
9	Using an artificial intelligence (AI) technique to develop an automatic AI prediction model for the collateral status of mCTA	Brain	Multiphase CTA	CNN	Prediction model reached an accuracy of 0.746 ± 0.008
10	Application of deep learning to evaluate Wilms tumor volume	Brain	MRI	nnUnet for tumor segmentation compared to manual segmentation	nnUnet: median Dice of 0.90, median HD95 of 7.2 mm. Deep learning shows potential to replace manual segmentation
11	Estimation of PSA level based on MR prostate image texture	Prostate	MRI	Classical texture analysis	Multiparametric classification using MIL-SVM: 92% accuracy
12	Detection of both exudates and hemorrhages in color fundus images	Eye	Non-mydriatic retinal camera	Unet for exudate and hemorrhage segmentation, YOLO5 for detection	Segmentation: Dice 85%; detection: 100%
13	Assessment of ER applications in the field of diagnostic imaging	All	USG, X-ray, CT	ER technologies	ER has significant potential to improve accuracy and efficiency in diagnostic imaging procedures and enhance the patient experience
14	Evaluation of three feature extraction methods and their performance in breast cancer diagnosis	Breast	Histopathology images	Three feature extraction methods: basic CNN transfer learning, VGG16, and a knowledge-based approach. All tested on standard classifiers	Accuracy: CNN: 85%, VGG16: 86%, knowledge-based (geometrical, directional, intensity features): 98%
15	More accurate classification of enhancing foci in MRIs of BRCA PV carriers for early breast cancer detection	Breast	MRI	CNN for tumor and non-tumor ROI detection	Correct classification of ~65% of tumors at an early time point
16	Multiclass segmentation and classification of liver images into lesions (6?) and normal tissue	Liver	Multiparametric MRI	nnUnet for segmentation, image registration, final UNet segmentation	Healthy liver/lesion classification: AUC ROC: 0.85, sensitivity and specificity: 0.79
17	Reducing image artefacts by inferring missing measurements using shape priors	Phantoms	CT	Deep convolutional GAN (DCGAN) architecture combining limited acquisition data and shape information	Improvement of reconstructed image quality by 7 dB Peak Signal-to-Noise Ratio compared to other methods
18	Classification of presence or absence of hemoperitoneum in USG images of Morrison’s pouch	Peritoneum	USG	Open-source DL provided by Google (https://teachablemachine.withgoogle.co, accessed on 1 March 2023)	External validation: sensitivity, specificity, and AUROC were 94%, 99%, and 0.97, respectively.
19	Denoising weighted filtered backprojection (wFBP) reconstructions	Phantom images, mouse scans	Photon-counting CT	Deep learning (UnetU) model for iterative reconstruction estimation from weighted filtered backprojection	UnetU provides higher SSIM PSNR when compared to classical ME NLM approach
20	Segmentation of liver portal veins from unlabeled H-phase and E-phase images by using the label of P-phase	Liver portal veins	CTA	Portal vein segmentation network (PVSegNet) applied to multiphase images	Portal vein segmented from H-phase and E-phase images achieved DSC 0.76 and 0.86 and Jaccard 0.61 and 0.76, respectively
21	Detection of active inflammation in the form of bone marrow edema (BME) in iliac and sacral bones	Sacroiliac joints	MRI	Own segmentation algorithm based on joint morphology	The Dice coefficient for automated bone segmentations with respect to reference manual segmentations was 0.9820
22	Comparison of the performance of path-finding algorithms for vessel labeling	Brain	3D TOF MRA	Three path-finding methods: depth-first search, Dijkstra’s, and A* algorithm	The best accuracy was observed using Dijkstra’s method
23	Quality comparison of two reconstruction algorithms of brain CT images	Brain	CT	DLIR (deep learning image reconstruction) and iterative reconstruction ASIR-V algorithms	DLIR shows superiority in both subjective and objective (SNR, CNR) assessments of image quality improvement
24	Building a 3D dose prediction model for glioblastoma VMAT treatment and testing its robustness and sensitivity for the purpose of quality assurance of automatic contouring	Brain	CT	Two-level cascaded 3D U-net trained for dose prediction	Improvement in dose and DVH score values, improvement in the spatial distribution of the predicted dose in the updated models at exactly the locations of concern
25	Overview of AI in radiology	All	All	Classical and deep ML	AI in radiology improves the quality of healthcare; however, several limitations exist
26	Quality comparison of two reconstruction algorithms of brain CT images	Brain	CT	Iterative (AIDR-3D) and deep learning-based (AiCE) reconstruction algorithms	AIDR-3D: lower artifact index, AiCE: higher CNR, lower median image noise, dependence on brain area
27	Object detection in chest X-ray images	Chest	Chest X-ray	YOLO v3 deep network	Average precision for 4 object classes: 0.815, APR: 0.987
28	Development of software tool for generation of MR synthetic images (various sequences)	Brain	MRI T1, T2, T2*, PD	Algorithm that modifies sequence parameters of input images	Qualitative visual assessment
29	Mitigation of motion-induced signal loss in liver DWI images	Liver	MRI DWI	Algorithm based on spatial scaling of average diffusion-weighted images	Reduced ADC bias, improved homogeneity of liver DWIs
30	Detection of lung cancer using histopathological images (LC25000 Dataset: Benign Lung tissue and Lung Adenocarcinomas)	Lungs	Optical histopathological images of lung and colon cancer cases	Kernel Fuzzy C-Means segmentation, Particle Swarm Optimization, and Grey Wolf Optimization for feature extraction/selection; classification based on classical ML models	Overall accuracy of 91.57% (in classifying benign and adenocarcinoma classes)
31	Reconstruction of a 3D tumor vascular network from histologic slices	Pancreatic ductal adenocarcinoma	Microscopic images of histological samples	Image registration (Fiji, Improved CWR), own vessel segmentation algorithm, 3D reconstruction by interpolation	Visual quality assessment of the obtained vascular network
32	Asymmetry detection in mammographic images for skin cancer detection support	Breast	Mammography	Dynamic Time Warping (DTW) for shape analysis; Growing Seed Region (GSR) method for breast skin segmentation	Accuracy of asymmetry detection: 83%, accuracy of skin segmentation: 66.7–90.5%
33	Simulation of respiratory lung motion and extraction of information for early diagnosis of lung cancer	Lungs	Chest X-ray	Combination of U-Net and a long short-term memory (LSTM) network for image generation and sequential prediction	prediction of respiratory motion: average Dice 0.96
34	Skin cancer classification (malignant vs. benign) in available datasets	Skin	Dermoscopic images	Ensemble of deep networks with different architectures	Classification accuracy: 93.18%, AUC: 0.932

## References

[1] Ozkara B., Chen M., Federau C., Karabacak M., Briere T., Li J., Wintermark M. (2023). Deep Learning for Detecting Brain Metastases on MRI: A Systematic Review and Meta-Analysis. Cancers.

[2] Rahmanuddin S., Jamil A., Chaudhry A., Seto T., Brase J., Motarjem P., Khan M., Tomasetti C., Farwa U., Boswell W. (2023). COVID and Cancer: A Complete 3D Advanced Radiological CT-Based Analysis to Predict the Outcome. Cancers.

[3] Nam H., Park S., Ho J., Park S., Cho J., Lee Y. (2023). Key-Point Detection Algorithm of Deep Learning Can Predict Lower Limb Alignment with Simple Knee Radiographs. J. Clin. Med..

[4] Mendes J., Matela N., Garcia N. (2023). Avoiding Tissue Overlap in 2D Images: Single-Slice DBT Classification Using Convolutional Neural Networks. Tomography.

[5] Rodrigues N., Silva S., Vanneschi L., Papanikolaou N. (2023). A Comparative Study of Automated Deep Learning Segmentation Models for Prostate MRI. Cancers.

[6] Ma H., Xu C., Nie C., Han J., Li Y., Liu C. (2023). DBE-Net: Dual Boundary-Guided Attention Exploration Network for Polyp Segmentation. Diagnostics.

[7] Zhang Y., Wu C., Xiao Z., Lv F., Liu Y. (2023). A Deep Learning Radiomics Nomogram to Predict Response to Neoadjuvant Chemotherapy for Locally Advanced Cervical Cancer: A Two-Center Study. Diagnostics.

[8] Lien W., Yeh C., Chang C., Chang C., Wang W., Chen C., Lin Y. (2023). Convolutional Neural Networks to Classify Alzheimer’ Disease Severity Based on SPECT Images: A Comparative Study. J. Clin. Med..

[9] Huang C., Chiang H., Hsieh C., Chou C., Jhou Z., Hou T., Shaw J. (2023). Using Deep-Learning-Based Artificial Intelligence Technique to Automatically Evaluate the Collateral Status of Multiphase CTA in Acute Ischemic Stroke. Tomography.

[10] Buser M., van der Steeg A., Wijnen M., Fitski M., van Tinteren H., van den Heuvel-Eibrink M., Littooij A., van der Velden B. (2023). Radiologic versus Segmentation Measurements to Quantify Wilms Tumor Volume on MRI in Pediatric Patients. Cancers.

[11] Gibala S., Obuchowicz R., Lasek J., Schneider Z., Piorkowski A., Pociask E., Nurzynska K. (2023). Textural Features of MR Images Correlate with an Increased Risk of Clinically Significant Cancer in Patients with High PSA Levels. J. Clin. Med..

[12] Farahat Z., Zrira N., Souissi N., Benamar S., Belmekki M., Ngote M., Megdiche K. (2023). Application of Deep Learning Methods in a Moroccan Ophthalmic Center: Analysis and Discussion. Diagnostics.

[13] Kukla P., Maciejewska K., Strojna I., Zapał M., Zwierzchowski G., Bąk B. (2023). Extended Reality in Diagnostic Imaging-A Literature Review. Tomography.

[14] Kode H., Barkana B. (2023). Deep Learning- and Expert Knowledge-Based Feature Extraction and Performance Evaluation in Breast Histopathology Images. Cancers.

[15] Anaby D., Shavin D., Zimmerman-Moreno G., Nissan N., Friedman E., Sklair-Levy M. (2023). ‘Earlier than Early’ Detection of Breast Cancer in Israeli BRCA Mutation Carriers Applying AI-Based Analysis to Consecutive MRI Scans. Cancers.

[16] Skwirczyński M., Tabor Z., Lasek J., Schneider Z., Gibała S., Kucybała I., Urbanik A., Obuchowicz R. (2023). Deep Learning Algorithm for Differentiating Patients with a Healthy Liver from Patients with Liver Lesions Based on MR Images. Cancers.

[17] Valat E., Farrahi K., Blumensath T. (2023). Sinogram Inpainting with Generative Adversarial Networks and Shape Priors. Tomography.

[18] Jeong D., Jeong W., Lee J., Park S. (2023). Use of Automated Machine Learning for Classifying Hemoperitoneum on Ultrasonographic Images of Morrison’s Pouch: A Multicenter Retrospective Study. J. Clin. Med..

[19] Nadkarni R., Clark D., Allphin A., Badea C. (2023). A Deep Learning Approach for Rapid and Generalizable Denoising of Photon-Counting Micro-CT Images. Tomography.

[20] Song G., Xie Z., Wang H., Li S., Yao D., Chen S., Shi Y. (2023). Segmentation of Portal Vein in Multiphase CTA Image Based on Unsupervised Domain Transfer and Pseudo Label. Diagnostics.

[21] Ożga J., Wyka M., Raczko A., Tabor Z., Oleniacz Z., Korman M., Wojciechowski W. (2023). Performance of Fully Automated Algorithm Detecting Bone Marrow Edema in Sacroiliac Joints. J. Clin. Med..

[22] Kim S., Kim Y. (2023). Effects of Path-Finding Algorithms on the Labeling of the Centerlines of Circle of Willis Arteries. Tomography.

[23] Pula M., Kucharczyk E., Zdanowicz A., Guzinski M. (2023). Image Quality Improvement in Deep Learning Image Reconstruction of Head Computed Tomography Examination. Tomography.

[24] Poel R., Kamath A., Willmann J., Andratschke N., Ermiş E., Aebersold D., Manser P., Reyes M. (2023). Deep-Learning-Based Dose Predictor for Glioblastoma-Assessing the Sensitivity and Robustness for Dose Awareness in Contouring. Cancers.

[25] Najjar R. (2023). Redefining Radiology: A Review of Artificial Intelligence Integration in Medical Imaging. Diagnostics.

[26] Cozzi A., Cè M., De Padova G., Libri D., Caldarelli N., Zucconi F., Oliva G., Cellina M. (2023). Deep Learning-Based Versus Iterative Image Reconstruction for Unenhanced Brain CT: A Quantitative Comparison of Image Quality. Tomography.

[27] Kufel J., Bargieł-Łączek K., Koźlik M., Czogalik Ł., Dudek P., Magiera M., Bartnikowska W., Lis A., Paszkiewicz I., Kocot S. (2023). Chest X-ray Foreign Objects Detection Using Artificial Intelligence. J. Clin. Med..

[28] Peretti L., Donatelli G., Cencini M., Cecchi P., Buonincontri G., Cosottini M., Tosetti M., Costagli M. (2023). Generating Synthetic Radiological Images with PySynthMRI: An Open-Source Cross-Platform Tool. Tomography.

[29] Raspe J., Harder F., Rupp S., McTavish S., Peeters J., Weiss K., Makowski M., Braren R., Karampinos D., Van A. (2023). Retrospective Motion Artifact Reduction by Spatial Scaling of Liver Diffusion-Weighted Images. Tomography.

[30] Shanmugam K., Rajaguru H. (2023). Exploration and Enhancement of Classifiers in the Detection of Lung Cancer from Histopathological Images. Diagnostics.

[31] Lindemann M., Glänzer L., Roeth A., Schmitz-Rode T., Slabu I. (2023). Towards Realistic 3D Models of Tumor Vascular Networks. Cancers.

[32] Bayareh-Mancilla R., Medina-Ramos L., Toriz-Vázquez A., Hernández-Rodríguez Y., Cigarroa-Mayorga O. (2023). Automated Computer-Assisted Medical Decision-Making System Based on Morphological Shape and Skin Thickness Analysis for Asymmetry Detection in Mammographic Images. Diagnostics.

[33] Yang D., Huang Y., Li B., Cai J., Ren G. (2023). Dynamic Chest Radiograph Simulation Technique with Deep Convolutional Neural Networks: A Proof-of-Concept Study. Cancers.

[34] Hossain M., Hossain M., Arefin M., Akhtar F., Blake J. (2024). Combining State-of-the-Art Pre-Trained Deep Learning Models: A Noble Approach for Skin Cancer Detection Using Max Voting Ensemble. Diagnostics.

[35] Strzelecki M., Badura P. (2022). Machine Learning for Biomedical Application. Appl. Sci..

[36] Piórkowski A., Obuchowicz R., Urbanik A., Strzelecki M. (2023). Advances in Musculoskeletal Imaging and Their Applications. J. Clin. Med..

[37] Strzelecki M., Kociołek M., Strąkowska M., Kozłowski M., Grzybowski A., Szczypiński P.M. (2024). Artificial intelligence in the detection of skin cancer: State of the art. Clinics in Dermatology.

[38] Mayerhoefer M.E., Materka A., Langs G., Häggström I., Szczypiński P., Gibbs P., Cook G. (2020). Introduction to Radiomics. J. Nucl. Med..

[39] Obuchowicz R., Kruszyńska J., Strzelecki M. (2021). Classifying median nerves in carpal tunnel syndrome: Ultrasound image analysis. Biocybern. Biomed. Eng..

[40] Nurzynska K., Piórkowski A., Strzelecki M., Kociołek M., Banyś R.P., Obuchowicz R. (2024). Differentiating age and sex in vertebral body CT scans–Texture analysis versus deep learning approach. Biocybern. Biomed. Eng..

